# Limited effects of action on ensemble processing

**DOI:** 10.3758/s13414-026-03319-3

**Published:** 2026-08-07

**Authors:** Shinhae Ahn, Richard A. Abrams

**Affiliations:** Department of Psychological and Brain Sciences, Washington University in St. Louis, Campus Box 1125, One Brookings Drive, St. Louis, MO 63130-4889 USA

**Keywords:** Action effect, Ensemble processing

## Abstract

Previous research has shown that production of an action toward a visual stimulus may prioritize features of that stimulus for a brief period of time. One recent report has suggested that such an *action effect* specifically influences ensemble processing – the extraction of summary statistics from a display of multiple items. In the present study we replicate the results that led to that conclusion by having participants assess the mean size of elements in an ensemble after having made an action to either a small or large object. The size of the acted-on object biased the ensemble judgment, but merely viewing the same object produced no such bias. In two subsequent experiments we found the same pattern of results when participants were asked to judge the size of an individual object – not an ensemble. Because the same pattern occurred even when ensemble processing was not required, the action appears to affect some aspect of the task other than ensemble processing (such as perception of visual stimuli in general, or processes related to memory, decision making or response selection), limiting the conclusions that are possible about ensemble processing, but revealing an important new effect of action.

## Introduction

There is a rich history of research on the relation between action and perception, beginning in the late nineteenth century (Woodworth, 1899). While much work has focused on the use of perceptual information in guiding actions (e.g., Woodworth, 1899), recent research has revealed a surprising effect of actions on perception: merely producing an arbitrary action (such as a key-press) in response to a stimulus can result in enhancement of features of that stimulus. For example, Buttaccio and Hahn (2011) showed a bias in visual search toward the color of a shape that had been responded to, but not merely observed, shortly before the search. Many other studies have shown a variety of different effects of action including enhancement of individual features of the acted-on object such as color and shape (Wang et al., 2021), an influence on the production of eye movements as well as manual responses (Wang et al., 2017), and strengthening of word-picture priming (Weidler & Abrams, 2018). According to one explanation, the stimuli that are present when an action is made are, by virtue of the production of the action, important, and deserve the benefit of prioritized processing (Weidler & Abrams, 2018). For example, people often produce repeated actions toward a given object (e.g., hammering a nail, or peeling a fruit), and any prioritization of the features of the acted-on object would presumably facilitate those repeated actions.

Recently it has been reported that actions may also influence the extraction of summary statistical information from a display of multiple objects – an ability known as *ensemble processing* (Knox et al., 2024). Ensemble processing is thought to play an important role in our ability to rapidly assess key attributes of complex scenes (Whitney & Yamanashi Leib, 2018). Knox et al. (2024) had participants judge the average size of an ensemble of eight oval shapes by selecting one of two alternative ovals as a response. Before viewing the ensemble, though, participants either made an action to a small or large rectangle, or they simply viewed a small or large rectangle. The key finding was that participants were more likely to incorrectly select a lure response alternative when that lure matched the (categorical: large vs. small) size of the earlier rectangle – but such a result only occurred when participants had produced an action – not when they had merely viewed the rectangle. The interpretation was that the action had biased attention in the processing of ensemble information to favor elements that were closer in size to the acted-upon rectangle. Such an influence of action on ensemble processing is potentially important because it contributes to a growing literature on the role of attention in ensemble perception (e.g., Chen et al., 2021; Kimchi & Sabary, 2025).

Although the conclusion offered by Knox et al. (2024) is consistent with an effect of action on ensemble processing, there are at least two other ways in which action might have affected responses in their task. First, if action biases processing of shapes congruent with the size of the acted-toward rectangle, then it is possible that the action affected the processing of the shapes that were presented in the response phase of the experiment as opposed to those in the ensemble itself. The response-alternative shapes were presented only 200 ms after removal of the ensemble, so any effect of action may still have been in force at that time. Second, action in the Knox et al. task may not have influenced perception at all – it may instead have influenced processes related to memory, decision making, or response selection. For example, after making an action to a large square, participants may simply have been biased to select the larger of the two response options. That the action effect might not have influenced perception in the task seems possible because the stimuli in the ensemble task (ovals) bore very little in common with the stimuli to which the actions were directed (rectangles). In past studies, action toward a stimulus of a particular color (Weidler & Abrams, 2014) or shape (Wang et al., 2021) were shown to bias attentional selection of items with matching colors or shapes. In the present study, the shapes to which actions were made (rectangles) were different from the shapes in the ensemble and response phases (ovals) and shared only the categorical feature of large or small size – and it is not clear if categorical size supports the action effect. Importantly, if either of these possibilities holds, then the results of Knox et al. (2024) cannot be taken to indicate an effect of action on ensemble processing.

There is one source of evidence that Knox et al. (2024) offered that might be inconsistent with the alternative interpretations just noted. In particular, in their Experiment 2, Knox et al. repeated the experiment just described but instead had participants make judgments about a single one of the shapes that were present in the ensemble display – not about a summary statistic. Importantly, this experiment included all of the other features of the task such as a response screen with two shapes and the requirement that participants select the response-screen shape that most closely matched the size of the target shape. If either of the alternative explanations noted earlier had been operating in their first experiment, then they might also have been expected to influence performance in Knox et al.’s Experiment 2; however, no such effect was seen. Instead, actions had no effect on responses in Experiment 2, on the surface ruling out an effect of action on perception of the response alternatives or on a bias to respond a certain way. Despite this, there is some reason to question the strength of the experiment as evidence against the alternatives being considered here. First, such a conclusion would be based on accepting the null findings of the lack of an action effect, and as Knox et al. noted, the Bayesian analysis that they conducted did not provide strong evidence in support of the null hypothesis. Second, the task used in the experiment was very difficult – with average accuracy only 60% when chance performance was 50%. This extremely low accuracy also limits the conclusions that are possible based on that experiment.

Considering the potential importance of an effect of action on ensemble processing, and given the alternative interpretations of the Knox et al. results that are feasible, we sought to provide a more strenuous test of the basic ideas relating action to ensemble processing. In Experiment 1, we replicated the main finding reported by Knox et al. suggestive of an effect of action on ensemble processing. In Experiments 2 and 3, we removed the ensemble and (in one experiment) altered aspects of the response requirements, and we still found the same effect of action, suggesting that the results reported by Knox et al. might not be related to ensemble processing at all – but instead reflect an influence of action on other aspects of the task.

## Experiment 1

Experiment 1 was designed to replicate the Knox et al. (2024) findings that revealed an effect of action on judgments about ensembles.

### Method

#### Participants

Forty-nine college students at Washington University in St. Louis (33 female; age range: 18–25 years) participated for course credit after reading an informed-consent document. Based on an effect size of *η*^2^ = 0.042 reported in the original experiment (Knox et al., 2024, Experiment 1) for the interaction effect of task type (action vs. viewing) and distractor congruency (congruent vs. incongruent to acted-on square size) on accuracy, we conducted an a priori power analysis using G*Power (Faul et al., 2007). This power analysis indicated that with a total sample size of 49 participants, the current study would have sufficient statistical power (power =.95) to detect an effect size of Cohen’s *f* =.214 (*η*^2^ = 0.042) for the interaction effect between task type and distractor-square size congruency. All participants reported having normal or corrected-to-normal visual acuity and normal color vision. The experimental procedure was approved by the Institutional Review Board of Washington University in St. Louis.

#### Stimuli and procedure

All stimuli were presented on an LCD monitor (1,680 × 1,050 pixels) with a gray background at a viewing distance of approximately 50 cm, controlled with PsychoPy software (Peirce, 2007).

**Fig. 1:**
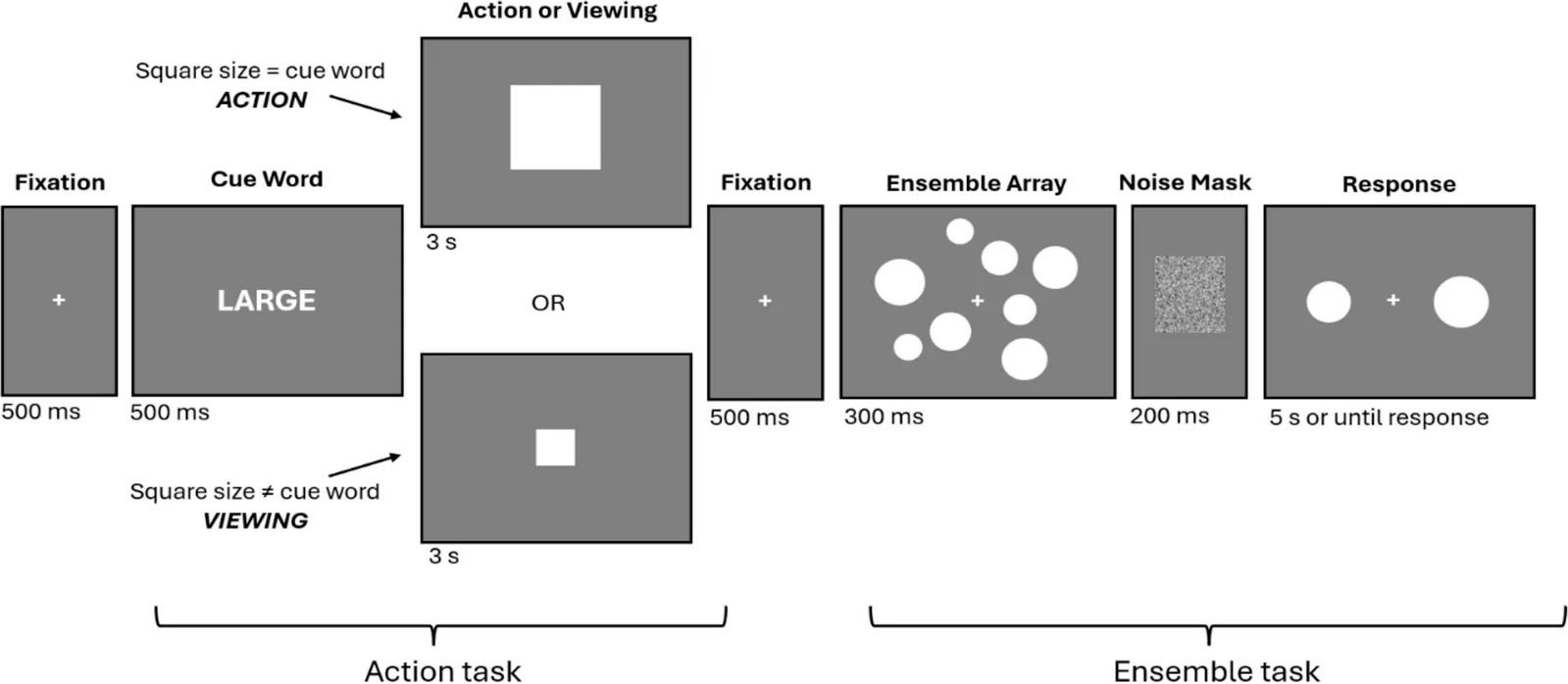
Sequence of events on a trial in Experiment 1. Each trial consisted of an action task and an ensemble task. In the action task, participants made an action (pressing the spacebar) if the size of the square matched the cue word; otherwise, they simply viewed the square. In the ensemble task, participants were asked to judge the average size of the circles in the ensemble array; the response was provided by selecting one of the two circles presented in the response display. See text for additional details

Figure 1 schematically illustrates the stimuli and procedure for the experiment. The experimental paradigm was the same as in Experiment 1 of Knox et al. (2024). Each trial consisted of two tasks: an action task followed by an ensemble task. The action task started with a fixation cross presented at the center of the screen for 500 ms, followed by a cue word (“LARGE” or “SMALL”) displayed for 500 ms. Either a large (8° × 8°) or small (2° × 2°) square was then presented on the screen for 3 s. Participants were instructed to press the spacebar (Action condition) as quickly as possible after square onset if the square size matched the cue word (e.g., a “LARGE” cue word and a large square). If the square size did not match the cue word, they were required to simply view the screen (Viewing condition) until it disappeared. Immediately after the action task, participants were presented with a set of eight circles selected from one of four possible ensemble sets with average diameters of 1.5° (range: 0.5–2.5°), 2.0° (range: 1.0–3.0°), 2.5° (range: 1.5–3.5°), or 3.0° (range: 2.0–4.0°). Within each ensemble set, circle sizes were evenly spaced to produce a gradual size variation across the specified range, without including the average size of each ensemble set and any overlapping individual sizes. The circles in the ensemble array were randomly placed at eight of 12 predetermined positions selected from two imaginary rings (10° and 20° diameters) where each ring contained six evenly spaced positions. Four positions were chosen randomly from each ring to form a final array of eight positions. The ensemble array was visible for 300 ms, and then followed by a 20° × 20° binary visual noise mask (element size = 0.0625°; phase-randomized on each trial) for 200 ms. After the mask, two response circles with different sizes appeared on the screen (one to the left and one to the right of center), one of which matched the average size of the ensemble task, while the other, the distractor, was either 0.25° larger than the maximum size or 0.25° smaller than the minimum size of the ensemble set. The size of the distractor was either congruent (Congruent condition) or incongruent (Incongruent condition) with the (categorical) size of the square from the action task. That is, if the square was large, a congruent distractor would be one that was 0.25° larger than the maximum size of the array. Participants selected the circle they judged to be closest to the average size of the circles presented in the ensemble by pressing the left or right arrow key.

#### Design

Ninety-six trials were presented in each of the four conditions from the factorial combination of the two factors (task type: action or viewing and distractor-square size congruency: congruent or incongruent size) in a random order, yielding a total of 384 trials that were divided into six experimental blocks. Within each set of 96 trials, each combination of cue word (LARGE or SMALL), square size (large or small), ensemble set (four different mean values), and correct response location (left or right) occurred equally often.

Participants completed 16 practice trials before beginning the main trials. The ensemble task followed only if participants made a correct response in the action task on a given trial. If the response in the action task was incorrect, the next trial began immediately. Participants were given a short break between each block and received feedback on their average accuracy and reaction time on the screen.

### Results

**Fig. 2:**
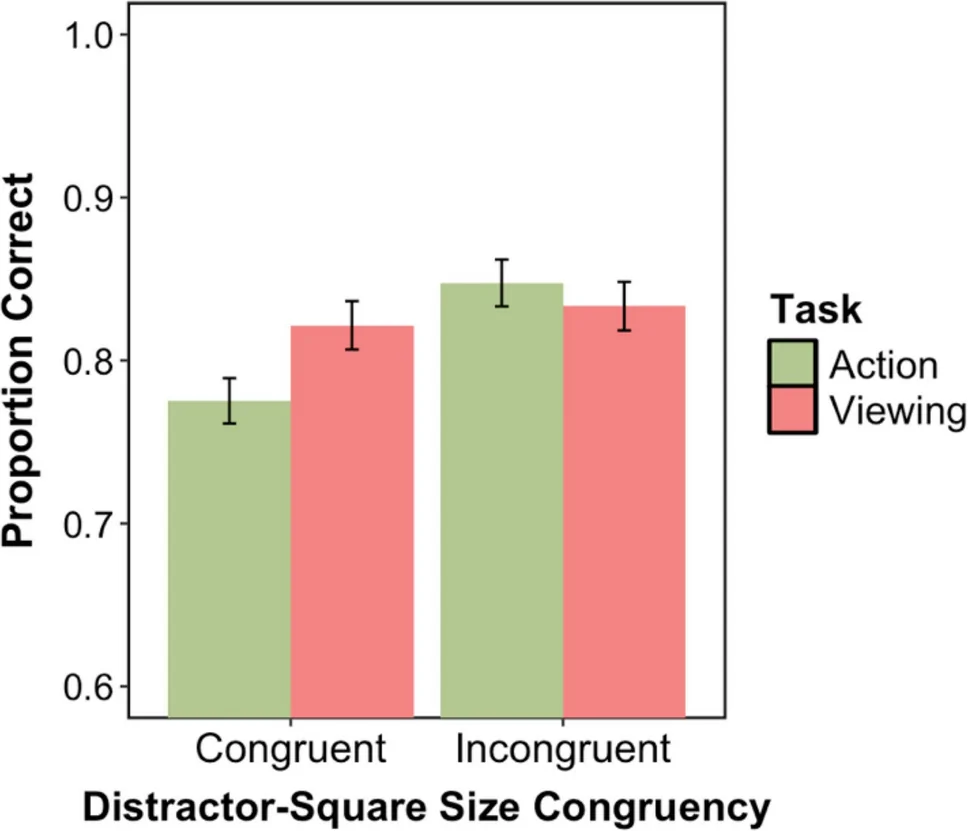
Results from the ensemble task in Experiment 1. The proportion of correct responses in the ensemble size estimation task is shown as a function of task type (Action vs. Viewing) and distractor-square size congruency (Congruent vs. Incongruent). When the distractor was congruent with the square, participants selected it more often, resulting in lower accuracy. That effect was enhanced in the Action task. Error bars represent within-subject standard errors

Only trials in which the action task was correctly completed and followed by the ensemble task were included in the data analysis (99.0% of trials). It is important to note that accuracy was the main variable of interest in the ensemble task rather than reaction time (RT), as it was not a speeded task. The results are shown in Fig. 2. A two-way repeated-measures analysis of variance (ANOVA) on accuracy in the ensemble task revealed a significant main effect of task type, *F*(1, 48) = 5.44, *p* =.024, *η*_p_^2^ =.10, showing that participants selected the average size of the ensemble set more accurately in the Viewing condition (82.7%) than in the Action condition (81.2%; when they made an action because the square size matched the cue word). There was also a significant main effect of distractor-square size congruency, *F*(1, 48) = 40.88, *p* <.001, *η*_p_^2^ =.46, showing that participants had lower accuracy when the distractor size in the test array was congruent with the square from the action task (79.8%) compared to when its size was incongruent with the square (84.1%). Importantly, a significant interaction effect between the two factors (task type and distractor-square size congruency) was found, *F*(1, 48) = 45.45, *p* <.001, *η*_p_^2^ =.49, replicating the previous findings in Knox et al. (2024).^1^

To further examine the accuracy difference between the Acting and Viewing conditions separately for the Congruent and Incongruent conditions, post hoc *t*-tests were conducted. For the Congruent condition, participants showed significantly lower accuracy in the Acting condition (77.5%) compared to the Viewing condition (82.1%), *t*(48) = 5.52, *p* <.001, Cohen’s *d* = 0.79, suggesting that acting on a square in the action task biased perception in favor of the size of the square. In contrast, for the Incongruent condition, the accuracy difference between the Acting (84.8%) and Viewing (83.3%) conditions was not statistically significant, *t*(48) = 1.77, *p* =.083, Cohen’s *d* = 0.25, also consistent with the idea that action biases perception toward the size of the acted on square (in the Incongruent condition, the distractor differed in size from the square, so the absence of a bias might be expected).

A two-way repeated-measures ANOVA on RT revealed no main effects of task type, *F*(1,48) = 0.002, *p* =.97, *η*_p_^2^ =.00 (631 ms for the Action condition; 633 ms for the Viewing condition), and distractor-square size congruency, *F*(1,48) = 0.07, *p* =.80, *η*_p_^2^ =.00 (633 ms for the Congruent condition; 631 ms for the Incongruent condition). An interaction effect between task type and distractor-square size congruency also was not statistically significant, *F*(1,48) = 2.88, *p* =.096, *η*_p_^2^ =.06.

### Discussion

The present findings replicate Experiment 1 of Knox et al. (2024). As in their experiment, we found that participants were more likely to select the incorrect response choice when it matched the (categorical) size of the square that had been presented earlier – but that only occurred when participants had made an action in response to the square, not when they had merely viewed it. The results are consistent with the interpretation that Knox et al. offered: the action toward the square biased processing of the subsequent ensemble in favor of elements similar in size to the square. That bias was revealed when the participants later indicated the perceived ensemble size at the end of the trial. Nevertheless, an alternative interpretation is also possible. According to the alternative, the action toward the square did not influence perceptual processing of the ensemble, but instead it influenced some other aspect of the task such as the memorial representation of the ensemble or processes related to decision making and response selection. For example, according to this alternative, when participants made an action toward a large square, they might have simply been more likely to select the larger of the two response options. Such an occurrence would produce exactly the same pattern of results that we (and Knox et al.) have reported – but the explanation would be very different – and, importantly, would have nothing to do with ensemble perception. We tested that possibility in our next experiment which was very similar to the present one but in which there was no ensemble judgment at all. If similar results are obtained, that would rule out any explanation based on altered ensemble perception.

## Experiment 2

The present experiment had many of the same components as Experiment 1: an action task, a critical stimulus display and a response phase. However, in the present experiment, the critical stimulus display consisted not of an ensemble of elements, but instead of only a single shape. If the action task influences judgments in the present task, then we can rule out the possibility that the action affected ensemble processing here – because there was no ensemble to process. As a result, the conclusion that ensemble processing was affected by the action in Experiment 1 would be substantially weakened.

### Method

#### Participants

As in Experiment 1, 49 college students at Washington University in St. Louis (35 female; age range: 18–22 years) participated for course credit after reading an informed-consent document. All participants reported having normal or corrected-to-normal visual acuity and normal color vision. The experimental procedure was approved by the Institutional Review Board of Washington University in St. Louis.

#### Stimuli, procedure, and design

**Fig. 3:**
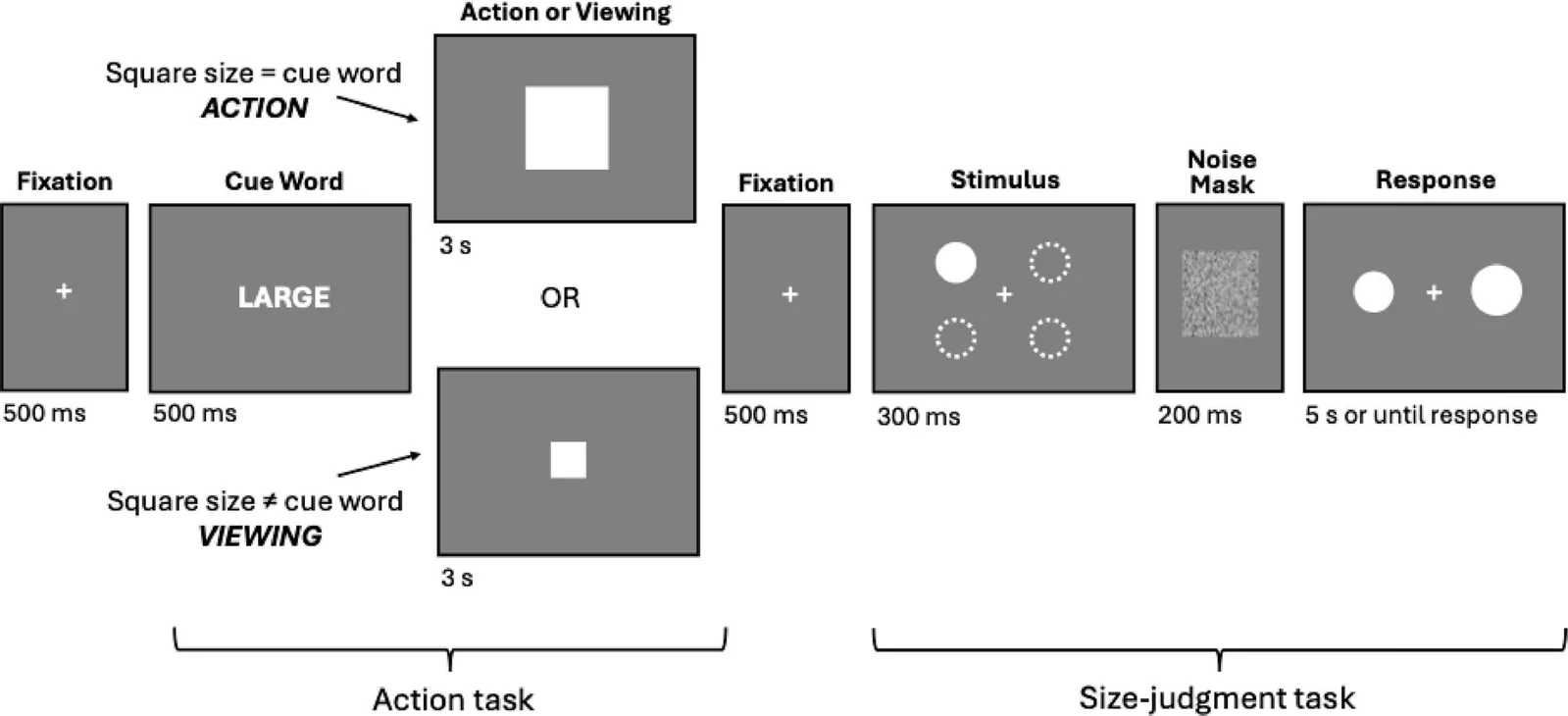
Sequence of events for the action task and size judgment task in Experiment 2. The action task was identical to that in Experiment 1. In the size judgment task, a single circle was presented at one of four locations. Participants were instructed to select, from two response options, the circle that matched the size of the target presented in the stimulus array

Details of the experiment are shown in Fig. 3. The experimental stimuli and procedure were similar to those of Experiment 1, except that after the action task, a single shape was presented for a size judgment, instead of presenting multiple shapes for an ensemble judgment.

In the size judgment task, each trial began with a 500-ms fixation cross, followed by a single target circle presented for 300 ms at one of four positions around the fixation cross, randomly selected on each trial. The size of this circle was randomly chosen from the average diameters (1.5°, 2.0°, 2.5°, or 3.0°) of the four possible ensemble sets from Experiment 1. After a 200-ms visual noise mask, two response circles with different sizes appeared on the screen. One circle matched the size of the previously presented target, while the other differed from the target size by $\pm 15\mathrm{\%}$ as a distractor. Participants were instructed to select the circle that matched the size of the circle presented in the stimulus array by pressing the left or right arrow key.

#### Design

The design was the same as in Experiment 1.

### Results

**Fig. 4:**
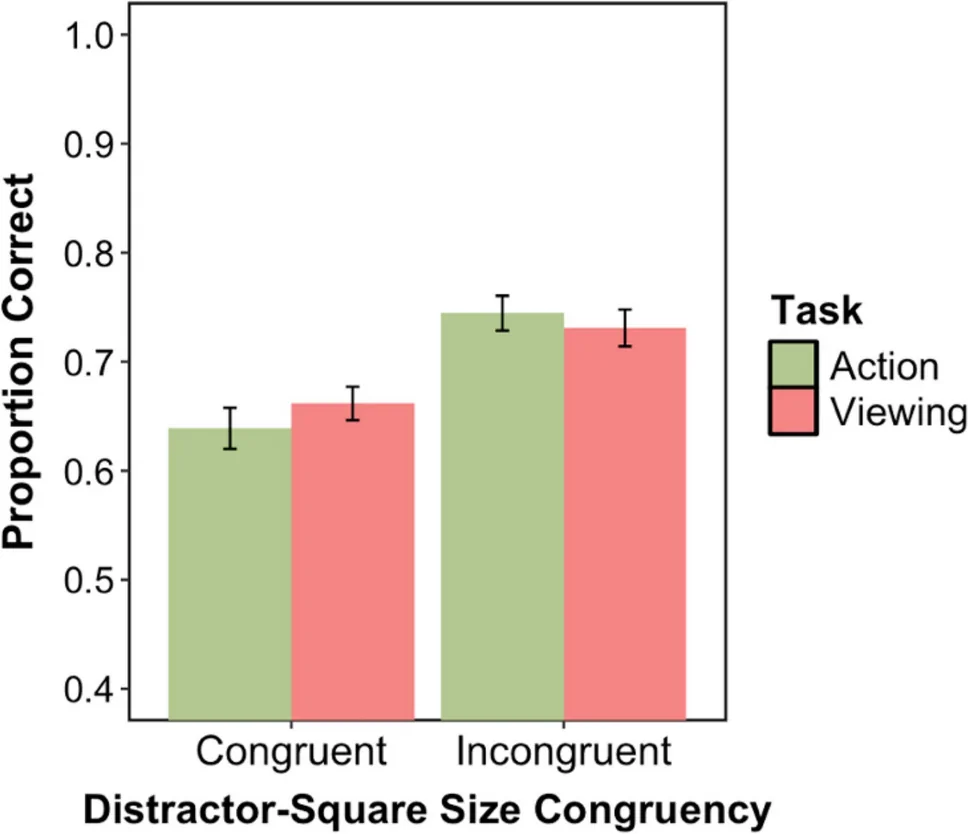
Results from the size judgment task in Experiment 2. The proportion of correct responses is shown as a function of task type (Action vs. Viewing) and distractor-square size congruency (Congruent vs. Incongruent). When the distractor was congruent with the square, participants selected it more often, resulting in lower accuracy. That effect was enhanced in the Action task. Error bars represent within-subject standard errors

As in Experiment 1, only trials in which the action task was correctly completed and followed by the size judgment task were included in the data analysis (98.6% of trials). The results of Experiment 2 are shown in Fig. 4. A two-way ANOVA on accuracy in the size judgment task revealed no main effect of task type, *F*(1, 48) = 0.64, *p* =.428, *η*_p_^2^ =.01. However, there was a significant main effect of distractor-square size congruency, *F*(1, 48) = 33.50, *p* <.001, *η*_p_^2^ =.41, indicating that accuracy was lower when the distractor size in the test array was congruent with the square from the action task (65.0%) than when it was incongruent (73.7%). Importantly, a significant interaction effect between the two factors was observed, *F*(1, 48) = 5.06, *p* =.029, *η*_p_^2^ =.10. As shown in Fig. 4, the congruency effect was larger in the Action condition than in the Viewing condition, suggesting that making an action increased the effect of congruency on the size judgment. This pattern replicates the findings from Experiment 1, even in the absence of an ensemble array. Post hoc *t*-tests showed that in the Congruent condition, participants showed significantly lower accuracy in the Action condition (63.9%) compared to the Viewing condition (66. 2%), *t*(48) = 2.05, *p* =.046, Cohen’s *d* = 0.29, consistent with action biasing the responses in favor of the size of the square. This difference was not statistically significant in the Incongruent condition (74.5% for Action; 73.1% for Viewing), *t*(48) = 1.58, *p* =.120, Cohen’s *d* = 0.23, also consistent with action producing a bias toward the acted-on square (in the Incongruent condition, the distractor differed in size from the square).

A two-way ANOVA on RT revealed that the main effect of distractor-square size congruency was statistically significant, *F*(1, 48) = 7.94, *p* =.007, *η*_p_^2^ =.14, indicating that participants responded more quickly in the Incongruent condition (658 ms) compared to the Congruent condition (672 ms). However, neither the main effect of task type, *F*(1, 48) = 1.29, *p* =.261, *η*_p_^2^ =.03, nor the interaction effect between task type and distractor-square size congruency, *F*(1, 48) = 0.71, *p* =.403, *η*_p_^2^ =.02.

### Discussion

The present experiment revealed an effect of action similar to what we found in Experiment 1: producing an action toward a large or small square biased a subsequent judgment about the size of a test stimulus. However here, unlike in Experiment 1, the test stimulus was an isolated circle – not an ensemble – so the results cannot have anything to do with ensemble perception. Instead, the action presumably influenced either perceptual processing of the circle, or some other aspect of the task related to memory, decision making, or response selection.

## Experiment 3

In this experiment, we sought additional evidence of an effect of action on judgments about an individual stimulus. We reasoned that the two-alternative forced-choice (2AFC) responses in Experiments 1 and 2 might have played a role in the results obtained there. In particular, if action did indeed bias attention to the categorical size of the action cue (the square), and if that bias persisted until the display of the response alternatives, then a bias in selecting the response could explain the pattern of results. To rule out that possibility, in the present experiment we repeated Experiment 2, but we changed the nature of the response. Here, instead of presenting participants with a choice of two possible responses (as in Experiments 1 and 2, and in Knox et al., 2024), we presented a single shape and asked participants to adjust the size of the shape so that it matched the to-be-judged element from the critical stimulus display. By using only a single shape in the response phase, we could be sure that any influence of the action in this experiment could not be attributed to biased perception of one of two alternatives presented during the response phase.

### Method

#### Participants

As in Experiments 1 and 2, 49 college students at Washington University in St. Louis (26 female; age range: 18–24 years) participated for course credit after reading an informed-consent document. All participants reported having normal or corrected-to-normal visual acuity and normal color vision. The experimental procedure was approved by the Institutional Review Board of Washington University in St. Louis.

#### Stimuli and procedure

**Fig. 5:**
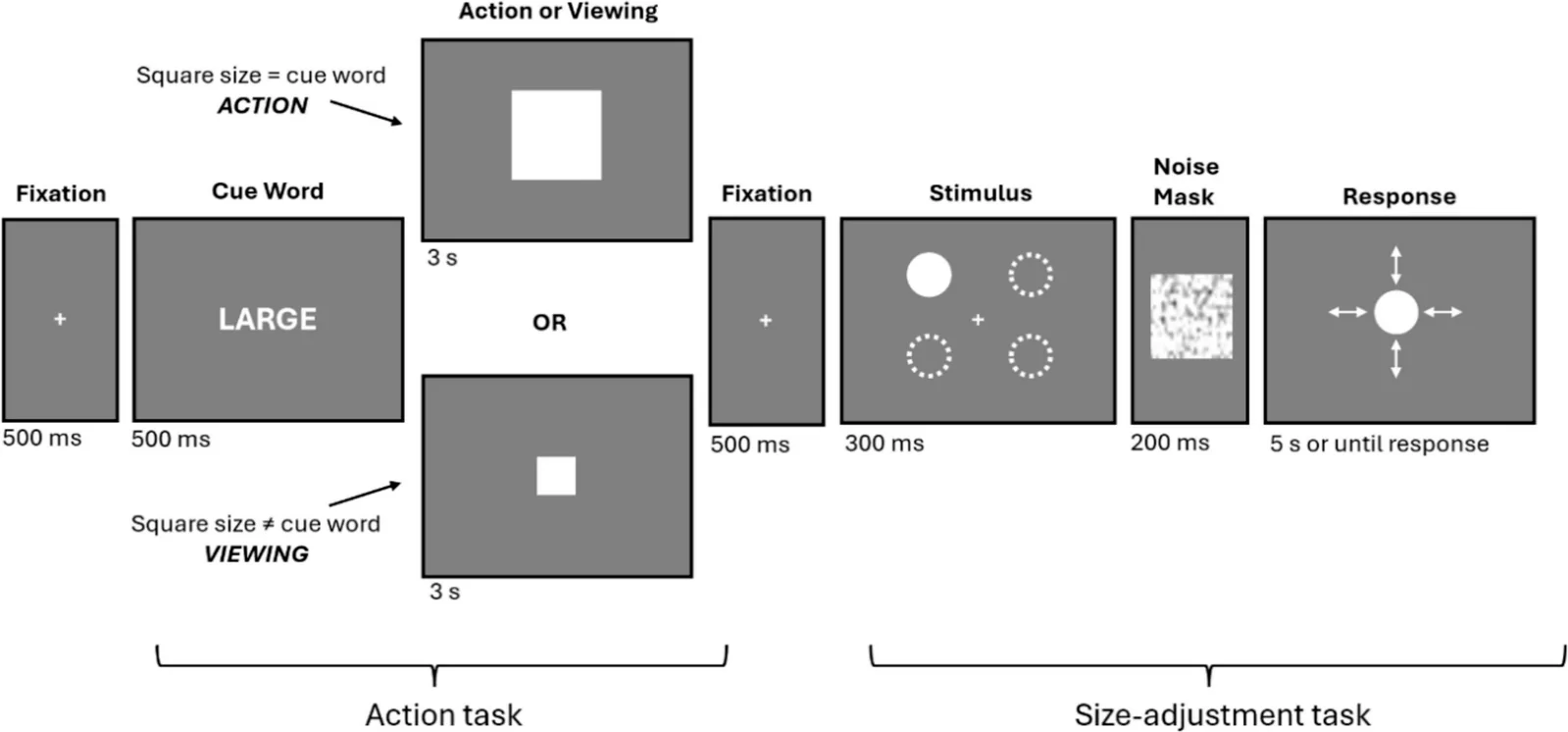
Sequence of events for the action task and size adjustment task in Experiment 3. The action task was identical to that in Experiment 1. In the size adjustment task, a single circle was presented at one of four locations (the dashed lines in the figure were not visible in the experiment). Participants adjusted the size of the response circle to match the remembered size of the stimulus (the arrows in the figure were not visible in the experiment)

Details of the experiment are shown in Fig. 5. The experiment was very similar to Experiment 2 except for one key change: Instead of presenting a 2AFC task for reporting the judged size, we presented a single adjustable shape. In the size adjustment task, each trial began with a 500-ms fixation cross, followed by a single circle presented at one of four positions randomly selected around the fixation cross as the target. The size of this circle was the same as in Experiment 2 – it was randomly chosen from the average diameters (1.5°, 2.0°, 2.5°, or 3.0°) of the four possible ensemble sets from Experiment 1. After a 200-ms visual noise mask, another single circle was presented at the center screen. The diameter of this response circle was randomly selected from a range of $\pm 75\mathrm{\%}$ around the diameter of the initial target circle (i.e., initial target size – (initial size × 0.75) to initial size + (initial size × 0.75)). Participants were required to adjust the size of the response circle to match that of the stimulus circle by using the left and right arrow keys for size adjustments and pressing the down-arrow key to record their response.

#### Design

The design was the same as in Experiments 1 and 2.

### Results

**Fig. 6:**
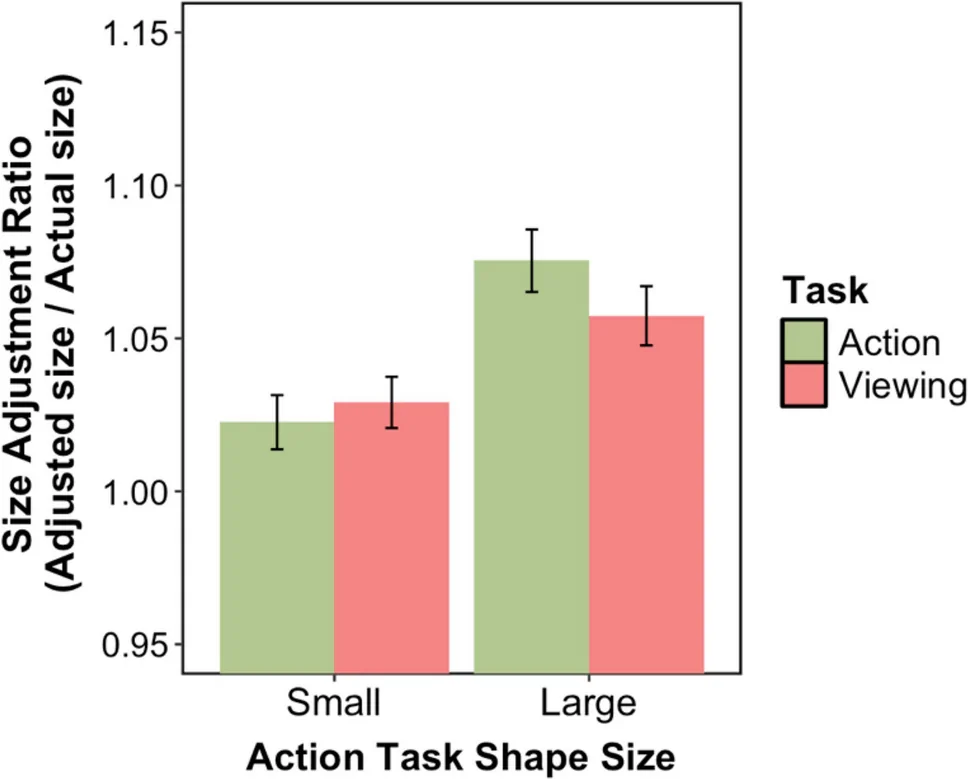
Results from the size adjustment task in Experiment 3. The size adjustment ratio (adjusted size of the test circle/actual target size) is shown as a function of task type (Action vs. Viewing) and acted-on square size (Large vs. Small). Error bars represent within-subject standard errors

As in Experiments 1 and 2, only trials in which the action task was correctly completed and followed by the adjustment task were included in the data analysis (98.7% of trials). The results of Experiment 3 are shown in Fig. 6. The main variable of interest was the size adjustment ratio, calculated by dividing each participants’ adjusted circle size in the response phase by the actual target size in the stimulus display. A ratio greater than 1.0 indicates that participants overestimated the target size, and larger values indicate larger judged target sizes.

A two-way ANOVA on the size adjustment ratio in the adjustment task revealed no main effect of task type, *F*(1,48) = 3.85, *p* =.056, *η*_p_^2^ =.07, but the main effect of square size was statistically significant, *F*(1,48) = 156.17, *p* <.001, *η*_p_^2^ =.77, showing that participants’ size adjustments were systematically influenced by the size of the square in the action task. Importantly, a significant interaction effect between the two factors was observed, *F*(1,48) = 22.28, *p* <.001, *η*_p_^2^ =.32, indicating that the square size had a greater effect on the response after an action was made compared to after only viewing of the square. Post hoc *t*-tests revealed that for the Large condition, participants adjusted the test circle to be larger in the Acting condition (ratio = 1.08) compared to the Viewing condition (ratio = 1.06), *t*(48) = 4.07, *p* <.001, Cohen’s *d* = 0.58, showing an influence of the action that favors the size of the square. In contrast, in the Small condition, there was no significant difference in size adjustments between the Acting (ratio = 1.02) and Viewing conditions (ratio = 1.03), *t*(48) = 1.93, *p* =.059, Cohen’s *d* = 0.28, but the difference is in the direction of an action-induced bias toward the size of the square.

A two-way repeated-measure ANOVA on RT revealed a significant main effect of task type, *F*(1,48) = 14.97, *p* <.001, *η*_p_^2^ =.24 (2.4 s for the Action condition; 2.5 s for the Viewing condition), indicating that participants responded more quickly in the Action condition compared to the Viewing condition. However, neither the main effect of square size, *F*(1,48) = 0.99, *p* =.324, *η*_p_^2^ =.02, nor the interaction effect between task type and square size, *F*(1,48) = 2.03, *p* =.160, *η*_p_^2^ =.04, were statistically significant.

### Discussion

In the present experiment, as in the previous experiments, producing an action to a square biased the judgment of the size of a subsequently presented stimulus. Here, as in Experiment 2, only a single circle (not an ensemble) was presented, so the results do not reflect anything about ensemble perception. In addition, the response display in the present experiment contained only a single adjustable-size circle (unlike in the earlier experiments), so the effect of action could not have been to bias the processing in favor of one of two presented response alternatives (because there was only one circle present, not two).^2^

## General discussion

The present experiments have revealed a new influence of action on cognition, while at the same time offering a new interpretation of earlier findings related to action. In Experiment 1 we showed that people were more likely to erroneously select one of two different-sized response alternatives when they had recently produced an action to a similar-sized shape. The response was provided as a judgment of the mean size of an earlier ensemble of shapes. As a result, it is not clear if the action affected processing of the ensemble, memory of the ensemble, processing of the response alternatives, or decisions about the response. Experiments 2 and 3 clarified the situation by showing that similar results could be obtained when there was no ensemble to process—participants there were asked to judge only a single isolated shape. In Experiment 2, participants reported the remembered size of the target circle by choosing the closest one of two response circles. In Experiment 3, participants reported the remembered size by adjusting the size of a response circle. In both experiments, an earlier action to a large shape caused participants to be more likely to select a larger response alternative (Experiment 2) or produce larger adjusted responses (Experiment 3), a result that cannot stem from any change in ensemble processing. Instead, the results suggest that action either affected perceptual processing of the isolated circle or of the response display, or it influenced some other aspect of the task such as memory for the stimulus or decision making or response selection. The findings not only reveal a new effect of action, but they also substantially weaken claims that action affects ensemble processing: Because the same effects of action were observed for isolated stimuli and for ensembles, there is no reason to propose any special impact of action on ensemble perception.

### Categorical size is relevant to actions

The present findings (along with those of Knox et al., 2024) show a unique new dimension that is enhanced by an action. In particular, the categorically-specified size of an acted-toward object plays a role in subsequent processing. In past work on the action effect, researchers have shown that action can enhance the color (Buttaccio & Hahn, 2011; Weidler & Abrams, 2014), shape (Wang et al., 2021), and meaning (Weidler & Abrams, 2018) of an acted-on stimulus. We can now add categorical size as an additional dimension that is enhanced by action. Taken together, the widespread effects of action reveal an important role of action in prioritizing the stimuli in our environment.

### Potential loci of the effect

Despite the new finding that actions can in some way prioritize the categorical size of an acted-toward object, there is still much that is not known about the precise locus of the effect. One possibility is that action to a shape actually shifted the perception of the size of subsequent stimuli in the direction of the size of the acted-toward object. In fact, a similar result has been shown to occur for attributes such as orientation in studies of *serial dependence* in vision. For example, in the experiments by Fischer and Whitney (2014), judgments of the orientation of a stimulus were shifted in the direction of the orientation of a previously presented stimulus. Similar results were reported by Ceylan and Pascucci (2023) and Pascucci et al. (2019). In those studies it was found that the influence of an earlier stimulus may depend upon whether a response had been required to that stimulus: Perception was shifted toward previous stimuli that had required a perceptual judgment but away from stimuli that were to be ignored. These results correspond well to the patterns that have been reported involving the action effect: When an action is made (compared to when one is withheld) there is an attentional or perceptual prioritization of certain attributes of the acted-toward object. While serial dependence has been shown for a variety of attributes (see Pascucci et al., 2023, for a review), it has not yet been demonstrated for categorical size, so it is not clear if the present findings reflect the operation of the same mechanism.

A related possibility is that action does not explicitly prioritize attributes of the acted-on object, but instead increases the probability that the object will be integrated with subsequent stimuli.^3^ For example, action toward a large square might cause the large shape to be integrated with the subsequent test stimulus in perceptual assessment, biasing the reported size of the test stimulus to be somewhat larger. Importantly, such an effect, if it occurred, appears to occur for individual stimuli – not just ensembles.

It is also possible that the present findings stem from an action-induced bias that is not perceptual. One possibility is that action influenced the remembered representation of the target stimulus, or some processes associated with decision making in selecting the response on a trial. For example, some researchers have shown that experimental demand characteristics may play a role in some perceptual judgments (e.g., Durgin et al., 2009). Whatever the locus of the effect, it is clear that it is not restricted to ensembles.

### Limits to effects of action on ensemble processing

Our results from Experiments 2 and 3 cannot be due to ensemble processing because there was no ensemble to process in those experiments. However, those findings do not definitively rule out a potential effect on ensemble processing in Experiment 1. That is, it is logically possible that in addition to an influence on perception of isolated shapes or other processes related to the selection of a response, as shown in Experiments 2 and 3, actions may also influence the processing of ensemble statistics as suggested by Knox et al. (2024), and presumably as revealed in the present Experiment 1. While we cannot definitively rule out that possibility here, our similar findings with (Experiment 1) and without (Experiments 2 and 3) ensembles substantially undermine any claim about specific effects of action on ensemble processing. Put simply, there is no reason to propose a role of action in ensemble perception given the identical effects in experiments without ensembles. More work will be needed before it will be possible to determine if indeed there are any unique effects involving ensembles. That work might be especially challenging because it will be necessary to eliminate any possible influence of the action on decision making and other processes related to response selection in any task used.

### Word versus shape

We have characterized the action effect here as arising from the correspondence between an action and the categorical size of the shape that was present at the time of the action. However, it is important to note that the categorical size indicated by the cue word (LARGE or SMALL) was completely confounded with the size of the acted-upon square. That is, whenever an action was made to a large square, the word LARGE had just recently been presented. Thus, it is logically possible that the effect of action stems not from the size of the acted-on shape, but instead from the meaning of the cue word. Earlier studies have ruled out the meaning of the cue word as the sole basis of the action effect (Weidler & Abrams, 2014) but it is possible that the word played some role in the present experiments.

### Evidence against a perceptual effect

Although we do not know how long the effect of an action might persist, it would be possible to reach some important conclusions about the locus of the action effect in the present experiments if we could assume that the effect of action persisted from the moment of action until the end of the trial. If that were the case, we could definitively rule out any effect of action on perceptual processes. The reason for this follows the logic of the El Greco fallacy (as described by Firestone & Scholl, 2014). Consider the paradigm from Experiment 3. If action had influenced some perceptual aspect of the stimulus circle in the size adjustment task (*any* perceptual aspect), then, if the effect persisted until the end of the trial, the action would have influenced the same perceptual aspect of the response circle. As a result, a response circle that was physically identical to the stimulus circle would also be a perceptual match to it – and there would be no expectation that the earlier action should bias the adjusted size (or anything else) of the response circle. For example, imagine that an action to a large square does indeed cause subsequently presented circles to look 10% larger than their actual size. That would cause both the stimulus circle and the response circle to look 10% larger than their actual sizes. In that case, a response circle that was an exact physical match to the stimulus circle would be perceptually identical to the stimulus circle. Participants should adjust the response circle to be the same physical size as the stimulus circle, and as a result, the perceptual distortion would not be evident in the response. However, in our experiment, because the adjusted size of the response circle was indeed affected by the earlier action, we can confidently conclude that the effect of the action was not on perceptual processes, but instead on some other processes related to decision making or the selection of a response. Of course, such a conclusion is possible only if we can assume that the effect of the action persisted throughout the trial. At the present time, the duration of the action effect has not been systematically studied.

## Conclusion

The present results add to the growing literature that reveals the important role played by action in guiding aspects of cognition, perception, and decision making. We have shown that action appears unlikely to affect ensemble processing per se, but action leads to prioritization of the categorical size of the acted-on object, and may influence perception or memory for individual items or subsequent decision making and response selection.

## Data Availability

All data and study materials supporting the findings of this study are publicly available in the Open Science Framework (OSF) repository: https://osf.io/5fcp3/.

## Footnotes

1 We also compared the pattern of results between each of the four different mean ensemble sizes by conducting an ANOVA with mean ensemble size as a factor. The same task type by distractor-square size congruency interaction was found. Each ensemble size showed the same pattern reported here, with some showing the pattern more strongly, as revealed by a distractor-square size by congruency by ensemble size interaction.

2 Note that it might be argued that the action biased perceptual processing of the single response circle. For example, after making an action to a large square, the bias might have somehow enhanced the perceptual strength of large circles, causing participants to be more likely to select a large circle as matching the size of the test circle. Such an effect would be difficult to distinguish from a simple bias in response that does not have a perceptual basis. We discuss this further in the *General discussion*.

3 We thank an anonymous reviewer for suggesting this possibility.

## Code availability

All experimental and analysis code supporting the findings of this study is publicly available in the Open Science Framework (OSF) repository: https://osf.io/5fcp3/.

## Declarations

### Open practices statement

All data, study materials, and analysis code supporting the findings of this study are publicly available in the Open Science Framework (OSF) repository: https://osf.io/5fcp3/.
